# Association of serum levels of soluble triggering receptor expressed on myeloid cells-1 with endothelial dysfunction in patients with type 2 diabetes

**DOI:** 10.3389/fcdhc.2025.1555655

**Published:** 2025-12-10

**Authors:** Wenwen Kong, Wenjun Sha, Jun Lu, Tao Lei

**Affiliations:** 1 Shanghai Putuo Central School of Clinical Medicine, Anhui Medical University, Shanghai, China; 2 Fifth Clinical Medical College, Anhui Medical University, Anhui, China; 3 Department of Endocrinology, Putuo Hospital, Shanghai University of Traditional Chinese Medicine, Shanghai, China

**Keywords:** patients with type 2 diabetes mellitus, flow-mediated dilation, endothelial function, TREM-1, diabetic

## Abstract

**Background:**

The correlation between TREM-1 and vascular complications in patients with type 2 diabetes was a subject of debate. This study aimed to investigate the potential correlation between TREM-1 and flow-mediated dilatation (FMD) in patients with type 2 diabetes mellitus.

**Methods:**

In this retrospective cohort research, 201 patients with type 2 patients diabetes were enrolled. The FMD Vascular Endothelial Cell Function Test Instrument was used to evaluate endothelial dysfunction. The serum levels of TREM-1 were measured using enzyme-linked immunosorbent assay. The Spearman correlation test was employed to determine the association between TREM-1 and FMD. Univariable logistic regression analysis was conducted to assess the relationship between TREM-1 and FMD. Additionally, receiver operating characteristic curve analysis was used to determine the TREM-1’s predictive value. The statistical significance was evaluated using a two-tailed P-value >0.05.

**Results:**

The study involved dichotomizing diabetic patients into low FMD (n = 138) and high FMD (n = 63) groups. The results showed that serum TREM-1 levels were significantly higher in the low FMD group than in the high FMD group (33.6 vs 58.0 pg/ml, P<0.001). A univariate logistic regression analysis revealed a statistically significant association between FMD and TREM-1 (P<0.05). The area under the curve for the receiver operating characteristic curve for model 1 (TREM-1) analysis was 0.66 (0.58-0.74) (P 0.001). Using the criteria of maximal Youden index, the threshold value for TREM-1 was found to be 38.16 ng/ml. This value showed a sensitivity of 75.4% and a specificity of 54% in predicting endothelial dysfunction in patients with type 2 diabetes mellitus.

**Conclusion:**

Serum TREM-1 levels were associated with FMD, indicating that TREM-1 could be a valuable biomarker for assessing endothelial function in T2DM patients.

## Introduction

As society has progressed and changed, diets and lifestyles have changed profoundly, leading to a significant increase in the prevalence of patients with diabetes. Over the past decade, the population of diabetic adults has surged remarkably by 56%, with figures escalating from 90 million to an estimated 140 million individuals between approximately 2011 and 2021. Diabetes is a metabolic disease that affects the entire body and can cause severe damage to both large and small blood vessels (1), and recent research indicates that oxidative stress and inflammation are key factors in the development of endothelial dysfunction (2).

The definition of endothelial dysfunction varies depending on the organ studied; however, in general, endothelial dysfunction can be characterised by impaired endothelium-dependent dilatation to agonists, to shear stress, or to local ischemia. In the specific context of proliferative diabetic retinopathy (PDR), the dysfunction is characterised as endothelial activation with a pro-inflammatory and proliferative phenotype. This ultimately leads to the disruption of vascular integrity, resulting in increased permeability (3). Flow-mediated vasodilation (FMD) measurements are commonly used to assess endothelial function in the brachial artery (4), examining peripheral artery endothelium-dependent dilation with high-resolution ultrasonography. The dilation is largely a NO-mediated process in response to the sudden increase in blood flow or shear stress (5). Lower FMD values suggest impaired nitric oxide (NO) production or bioavailability, which often indicates the presence of endothelial dysfunction (6). In 1989, Anderson published a seminal paper introducing FMD measurement methods in people (7), followed by Celermajer’s application to clinical research in 1992 (8). Several studies have shown endothelium dysfunction to be predictive of cardiovascular events (9).

The triggering receptor expressed on myeloid cells-1 (TREM-1) is an amplifier of the innate immune response in infectious and non-infectious inflammation. It is expressed in endothelial cells, neutrophils, and monocytes/macrophages (10). TREM-1 is activated by the DNAX activation protein of 12 kDa (DAP12) (11), Once activated, TREM-1 induces the production and release of pro-inflammatory cytokines and chemokines, in addition to increasing its own expression and circulating levels of the cleaved soluble extracellular portion of TREM-1 (sTREM-1), and plays a crucial role in chronic inflammation pathogenesis (12). Patients who experienced a heart attack alone had lower serum TREM-1 levels than those who had both diabetes and a heart attack (13). In a multicenter cohort study of individuals diagnosed with acute coronary syndrome, it was found that the sTREM-1 gene was a significant and independent predictor of major adverse cardiovascular events (14). Additionally, a study conducted in Denmark found that children diagnosed with type I diabetes had significantly higher levels of the sTREM-1 gene compared to their healthy siblings (15).

This study aimed to investigate the relationship between vascular endothelial function and serum TREM-1 levels in patients with T2DM. To evaluate the endothelial function of diabetic patients, FMD was examined. Serum TREM-1 levels were determined using ELISA (16).

## Methods

### Study design and participants

This cross-sectional study was conducted in a single center at Putuo hospital, which was affiliated to the Shanghai University of Traditional Chinese Medicine. A total of 201 patients diagnosed with type 2 diabetes between January 2021 and April 2023 were included in the study. Individuals with the following conditions: (1) patients with type 1 diabetes mellitus, gestational diabetes, or any other form of diabetes; (2) acute complications related topatients patients with type 2 diabetes (n=10); (3) signs of serious underlying illnesses, such as malignancies, cardiovascular disease by instability, and renal and liver dysfunction were excluded.

The study procedure was approved by the institutional review board at Shanghai University of Traditional Chinese Medicine’s Putuo Hospital. In accordance with this protocol, all participants provided their informed consent by signing a consent form.

### Anthropometric and laboratory measurements

When participants were admitted, anthropometric data and biochemical parameters were assessed. Calculating the Body Mass Index (BMI) requires dividing weight by meters squared (kg/m2). HOMA-IR was calculated using the formula FBG(mmol/L)*FI(mu/ml)/22.5 (17). Following the Chinese Diabetes Society’s recommendations for diabetic diets (18). blood samples were collected from each patient the subsequent morning after a fasting period of at least ten hours. The Beckman Coulter AU5800 Automated Biochemistry Analyzer was utilized to analyze various biochemical markers, such as total cholesterol, triglyceride levels, fasting C-peptide, postprandial glucose, and fasting glucose. HbA1c levels were determined utilizing glycated hemoglobin analytical methods through the Tosoh Automated Glycohemoglobin Analyzer HLC723G11 from Shunan, Japan. Measurement of TREM-1 was performed with an ELISA kit supplied by Boster, China.

### Measurement of FMD

A semi-automated device for FMD measurement was used by an experienced physician (UNEXEF, UNEX, Nagoya, Japan). Patients must fast for at least four hours and refrain from using tobacco, alcohol, or caffeine for at least 12 hours prior to FMD. Participants were instructed to assume a supine position for a duration of 15 minutes in a tranquil, dimly lit, room with a temperature control of 22-25 °C. The blood pressure cuff was positioned approximately two centimeters below the antecubital fossa on the right forearm. Subsequently, a baseline image of the right brachial artery was captured using the probe. In order to induce an elevation in systolic blood pressure, the cuff was inflated to achieve an increase of 50 mm Hg. The cuff was subsequently deflated, and the brachial artery’s maximal diameter upon cuff release was noted. The formula for calculating FMD is: (maximum diameter – baseline diameter) / baseline diameter × 100%, which represents the percentage of maximum dilation.

### TREM-1 measurement

Serum levels of TREM-1 were measured using a TREM-1 ELISA kit (BOSTER, China) following the manufacturer’s instructions. The results were presented in pg/ml.

### Statistical analysis

The Kolmogorov-Smirnov test was used to assess the distribution of continuous variables. The frequency (%) was used to display categorical variables. Normally distributed continuous variables were expressed using the mean and standard deviation, while continuous variables with abnormal distributions were expressed using the median (interquartile range [IQR]). In the case of normally distributed continuous variables, the independent samples t-test was used for comparing two groups, while the Mann-Whitney U-test was used when the data distribution was skewed. Curve regression analysis, Spearman correlation analysis, and partial correlation analysis were used to evaluate the correlation between TREM-1 and FMD. The sensitivity and specificity of TREM-1 in predicting FMD in patients with type 2 diabetes were assessed through receiver operating characteristic (ROC) curve analysis. In all ROC curves, the optimal cut-off was determined using maximal Youden Index criteria. Data analysis was conducted using SPSS 23.0 software (IBM Corp., Armonk, NY, USA). Statistical significance was shown by a two-sided P < 0.05.

## Result

### Clinical characteristics of patients included

**Figure 1:**
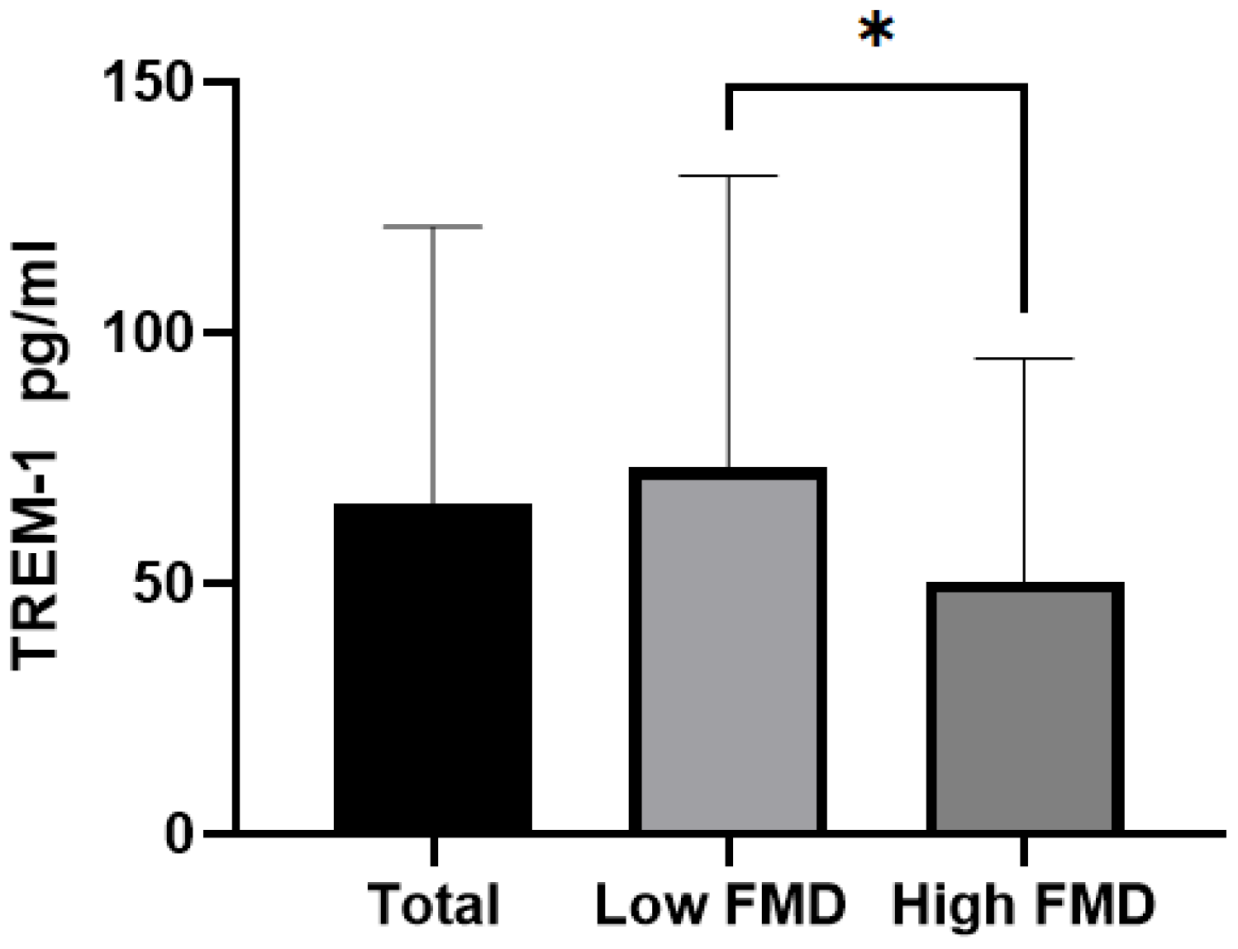
Comparison of TREM-1 levels among the Total group, low FMD group, and high FMD group. The bar chart shows the mean ± standard error (SE) of each group, with * indicating P <0.05.

The 201 diabetic patients were divided into two groups based on their FMD levels: low FMD (n=138, 1.4%~5.5%) and high FMD (n=63, 5.6%~8.4%). **Table 1** provides detailed information on the baseline characteristics and clinical parameters of these two groups. The low FMD group had older patients and higher TREM-1 levels compared to the high FMD group. Notably, the serum TREM-1 levels were significantly lower in the high FMD group than in the higher FMD group (33.6 pg/ml vs 58.0 pg/ml, P < 0.001). The bar chart in **Figure 1** more intuitively shows that the concentration of TREM-1 gradually decreases from the low FMD group to the high FMD group (P < 0.05).

**Table 1:** Baseline characteristics of patients.

Variables	Total ( n = 201 )	Low FMD ( n = 138 )	High FMD ( n = 63 )	P value
Gender (men), %	131(65.2)	93 (67.4)	38 (60.3)	0.33
Age, years	64 (56 – 70)	64 (56 – 71)	62 (55 – 67)	0.09
Diabetes duration, years	5 (0.33 – 18)	6.0 (0.25 – 20.0)	4.0 (1.0 – 15.0)	0.51
BMI, kg/m^2^	24.2 (22.0 – 27.1)	24.1 (22.0 – 27.0)	24.8 (21.8-27.8)	0.54
Current smoking, %	55 (0.27)	38 (27.5)	17 (27.0)	0.94
Alcohol drinking, %	54 (0.27)	39 (28.3)	15 (23.8)	0.51
Blood pressure				
SBP, mmHg	130 (123 – 143)	130 (124 – 145)	132 (120 – 143)	0.95
DBP, mmHg	82 (77 – 90)	82 (78 – 88)	84 (76 – 92)	0.35
HbA1c, %	9.50 (7.70 – 10.9)	9.50 (7.60 – 10.90)	9.60 (7.70 – 10.90)	0.95
FBG, mmol/L	7.90 (6.3 – 10.6)	7.90 (6.30 – 10.8)	8.00 (6.10-10.5)	0.84
120 min FPG, mmol/L	14.2 (10.6 – 18.3)	14.2 (10.9 – 18.1)	13.8 (10.2 – 19.0)	0.80
Fasting insulin, pmol/L	68.4 (42.3 – 109.9)	68.3 (41.1 – 109)	70.9 (42.3 – 118)	0.53
HOMA-IR	3.36 (1.94 – 5.86)	3.22 (2.01-5.71)	4.26 (1.86-7.29)	0.37
FCP, nmol/L	0.42 (0.23 – 0.68)	0.42 (0.22 – 0.69)	0.43 (0.25 – 0.68)	0.72
TC, mmol/L	4.82 (3.79 – 5.66)	4.90 (3.79 – 5.66)	4.54 (3.75 – 5.91)	0.70
TG, mmol/L	1.46 (1.05 – 2.13)	1.44 (1.05 – 2.12)	1.48 (0.98 – 2.37)	0.60
HDL-C, mmol/L	1.09 (0.91 – 1.28)	1.08 (0.91 – 1.28)	1.11 (0.94 – 1.29)	0.79
LDL-C, mmol/L	3.02 (2.27 – 3.8)	3.11 (2.29-3.83)	2.80 (2.25-3.80)	0.30
Free fatty acids, mmol/L	0.53 (2.27 – 3.8)	0.53 (0.40 – 0.67)	0.51 (0.31 – 0.67)	0.44
UACR, mg/g	16.3 (8.66 – 55.9)	18.9 (9.10 – 56.5)	14.6 (7.67 – 56.1)	0.28
TREM-1, pg/mol	52.86 (29.34 – 86.23)	58.0 (38.1 – 95.4)	33.6 (20.8 – 69.5)	<0.001

Data were expressed as median (interquartile range, IQR).

FBG, Fasting blood glucose; FI, Fasting insulin; PBG, Postprandial blood glucose; FCP, Fasting C peptide; SBP, systolic blood pressure; DBP, diastolic blood pressure; HbA1c, Hemoglobin A1c BMI, body mass index; TC, total cholesterol; TG, triglyceride; HDL-C, high-density lipoprotein cholesterol; LDL-C, low-density lipoprotein cholesterol; UACR, urine albumin creatine ratio; RBC, red blood cell; HB, hemoglobin; TREM-1, triggering receptor expressed on myeloid cells1.

HOMA-IR was calculated using the formula FBG(mmol/L)*FI(Mu/ml)/22.5 .

### Other indicators correlated with FMD

Based on the findings from **Table 2**, the Pearson correlation analysis revealed significant associations between FMD and both TREM-1 (P = 0.003) and triglycerides (P = 0.007). Conversely, no significant associations were observed between fasting plasma glucose, postprandial glucose, fasting C-peptide, systolic and diastolic blood pressure, body mass index, total cholesterol, HDL cholesterol, LDL cholesterol, and hemoglobin A1c. Following the adjustment for age and gender, the utilization of partial correlation analysis revealed a noteworthy association between TREM-1 and FMD.

**Table 2:** Parameters correlated with FMD as a continuous variable.

Variables	Pearson correlation		Partial correlation	
	r	P value	r	P value
Gender (men), %	0.043	0.542	–	–
Age, years	-0.079	0.266	–	–
Diabetes duration, years	-0.04	0.575	-0.018	0.797
BMI, kg/m^2^	0.071	0.316	0.052	0.470
TREM-1, pg/mol	-0.211	0.003	-0.202	0.004
HbA1c, %	-0.009	0.900	-0.018	0.803
FBG, mmol/L	0.070	0.326	0.070	0.325
120 min PBG, mmol/L	0.028	0.692	0.025	0.721
Fasting insulin, pmol/L	0.002	0.982	-0.001	0.986
HOMA-IR	0.050	0.480	0.045	0.532
FCP, nmol/L	-0.031	0.661	-0.022	0.753
TC, mmol/L	0.113	0.111	0.096	0.176
TG, mmol/L	0.188	0.007	0.176	0.013
HDL-C, mmol/L	0.026	0.713	0.030	0.669
LDL-C, mmol/L	-0.028	0.695	-0.046	0.522
Free fatty acids, mmol/L	0.097	0.171	0.095	0.182

FBG, Fasting blood glucose; PBG, Postprandial blood glucose; FCP, Fasting C peptide; SBP, systolic blood pressure; DBP, diastolic blood pressure; BMI, body mass index; TC, total cholesterol; TG, triglyceride; HDL-C, high-density lipoprotein cholesterol; LDL-C, low-density lipoprotein cholesterol; HbA1c, Hemoglobin A1c; TREM-1, triggering receptor expressed on myeloid cells1.

### Relationship between TREM-1 and endothelial dysfunction in patients with type 2 diabetes

**Figure 2:**
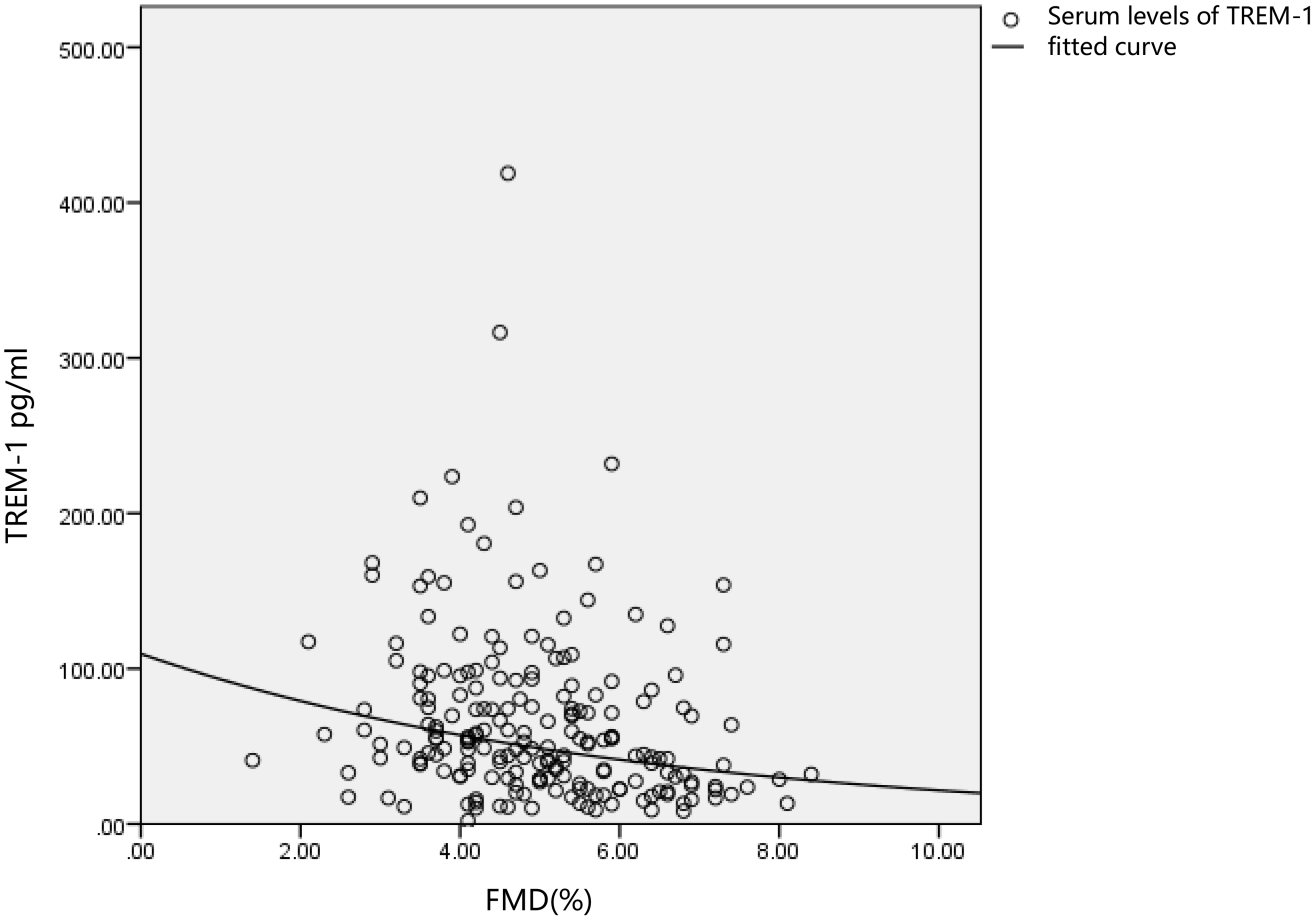
With measured FMD (%) in patients with type 2 diabetes plotted on the x-axis and serum TREM-1 levels on the y-axis. The circles in the figure represent serum TREM-1 levels, and the horizontal line represents the fitted curve between FMD (%) and TREM-1.

**Figure 2** shows that the correlation coefficient between TREM-1 and FMD (%) estimated by curve regression was 0.211, and the coefficient of determination (r²) was 0.065.

**Figure 3:**
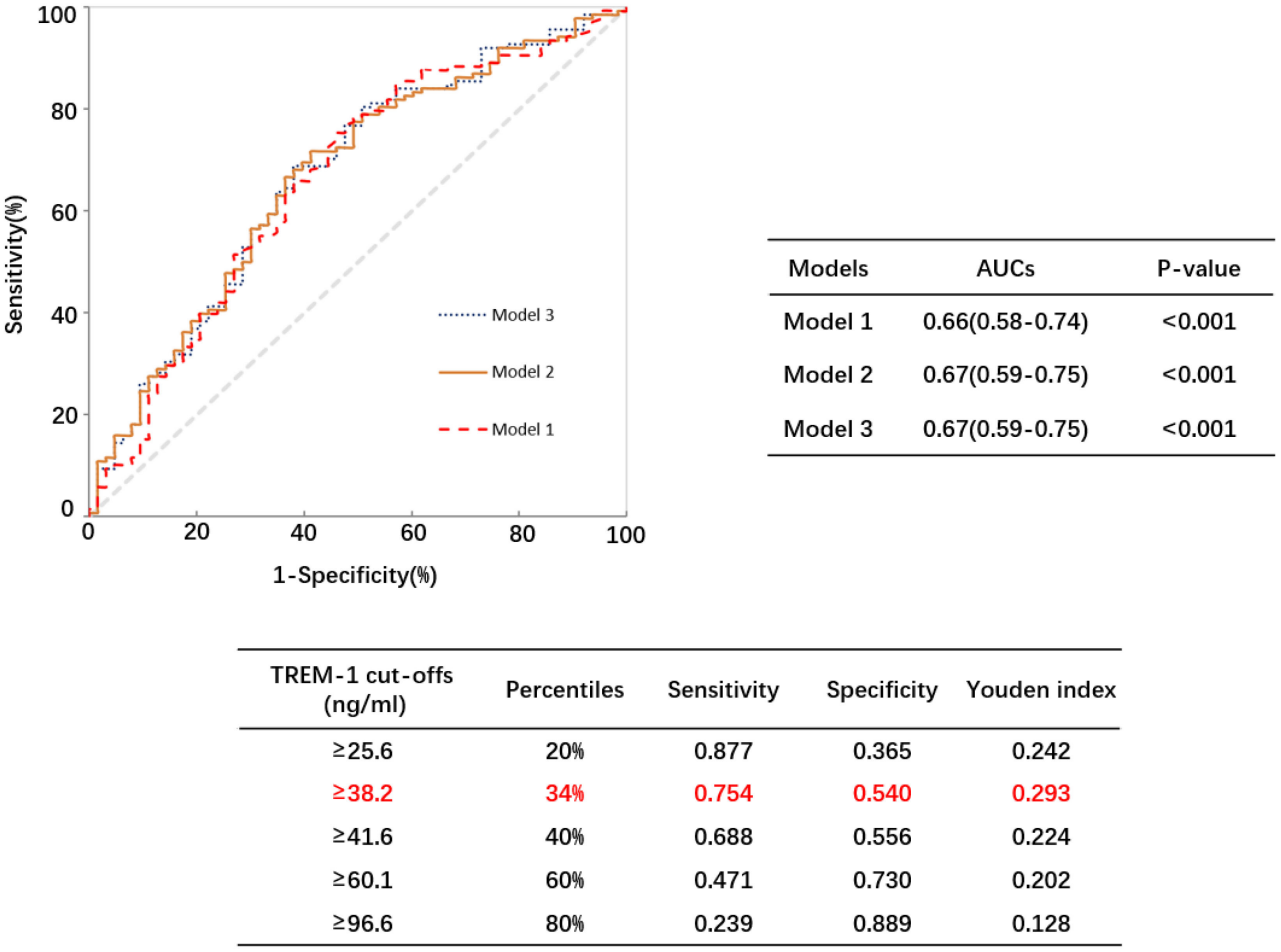
Receiver operating characteristic curves for TREM-1 predictive of endothelial dysfunction in patients with type 2 diabetes mellitus. AUC, area under the curve.

**Figure 3** illustrates that the area under the curve pertaining to model 1 (TREM-1) amounted to 0.66. Through ROC analysis, it was determined that the optimal critical value for TREM-1 in detecting endothelial dysfunction among patients with type 2 diabetes mellitus was 38.16 ng/ml. This critical value exhibited a sensitivity of 75.4% and a specificity of 54%.

## Discussion

The study found a negative association between serum TREM-1 levels and FMD in T2DM patients. Further ROC curve analysis indicated that TREM-1 showed good performance in the diagnosis of vascular endothelial dysfunction in type 2 diabetes.

As a transmembrane glycoprotein, TREM-1 is mainly expressed in endothelial cells, and monocytes/macrophages, and it is involved in both infectious and non-infectious diseases (19). TREM1-mediated amplification of proinflammatory immune responses worsens outcomes in models of infection such as sepsis (20) and non-infectious models, including rheumatoid arthritis (21), pancreatitis (22), atherosclerosis (23), and chronic obstructive pulmonary disease (24). Therefore, the potential mechanisms of TREM1 in endothelial dysfunction may involve the synthesis of inflammation amplification and subsequent oxidative stress imbalance. However, there have been few investigations into the relationship between TREM-1 and endothelial dysfunction in patients with type 2 diabetes. The research discovered that the expression of TREM-1 was significantly increased in endothelial cells. TREM-1 knockout specifically in endothelial cells alleviated BBB dysfunction and cognitive impairments (16). A positive correlation between TREM-1 expression and fasting plasma glucose, glycosylated hemoglobin, and the number of lesion vessels was discovered in another study (25). TREM-1 promotes inflammation and plaque instability by increasing the accumulation of subendothelial lipids and the production of pro-inflammatory cytokines and matrix-degrading enzymes (26). Wang’s research found that mice given pravastatin had lower levels of TREM-1 expression, as well as lower levels of IL-1β and TNF-α, two inflammatory factors (27). Additionally, a genetic study showed a strong correlation between TREM-1 gene polymorphisms and the severity of atherosclerosis in a Russian population (28). This data suggests that increased TREM-1 may represent endothelial inflammation in patients with atherosclerosis.

Endothelial dysfunction is a common chronic complication affecting most patients with T2DM.Through their effects on end-product composition, regulatory pathways, degree of inflammation, and the formation of atherosclerotic plaque, abnormalities in glucose metabolism predispose people to cardiovascular disease (29). Endothelial function, which includes vasoconstrictor-vasodilator and modulation anti-inflammatory as well as anticoagulant qualities, is essential for the preservation of vascular homeostasis. NO is an endothelium-derived mediator that inhibits cellular inflammation, and inflammatory cell adhesion suppresses thrombosis, and promotes blood flow in addition to limiting artery wall remodeling (30). *In vitro* studies on human endothelial cells have demonstrated that TREM-1 causes endothelial cell dysfunction by down-regulating the NO-related gene p-eNOS and up-regulating the production of TNF-α (31). One frequent test for endothelial function is FMD of peripheral conduit arteries. Diminished FMD is suggestive of reduced endothelial NO bioavailability and high levels of oxidative stress, which oxidize lipids and activate proinflammatory monocytes to cause atherosclerosis (32). A previous study has shown that FMD is lower in the T2DM group compared to the control group (33). In this prospective cohort, our data further confirms that TREM-1 is negatively correlated with FMD in patients with type 2 diabetes.

Our study provides new insights into the function of TREM-1 and its ability to predict endothelial dysfunction in individuals with type 2 diabetes. However, there are several limitations to this study. Firstly, the patient cohort was limited to a single center, which may not be representative of the broader patient population. Secondly, the grouping of FMD did not consider the potential influence of relevant medications. Finally, it is important to consider other confounding variables, such as the emotional and psychological state of the individuals involved.

## Conclusion

The study found a negative association between serum TREM-1 levels and FMD in patients with T2DM. TREM-1 may be a novel modulatory factor for metabolic disturbances in diabetic complications. Further analysis is needed to validate these findings and reveal the underlying mechanism of TREM-1 in the pathophysiology of endothelial dysfunction.

## Data Availability

The datasets presented in this study can be found in online repositories. The names of the repository/repositories and accession number(s) can be found in the article/supplementary material.
